# Complications associated with hyperextension bicondylar tibial plateau fractures: a retrospective study

**DOI:** 10.1186/s12893-021-01215-1

**Published:** 2021-06-25

**Authors:** Guoyun Bu, Weitang Sun, Yandong Lu, Meng Cui, Xi Zhang, Jie Lu, Jinli Zhang, Jie Sun

**Affiliations:** 1 Department of Traumatology, Tianjin Hospital, Liberation Road 406, Hexi District, Tianjin, 300000 China; 2 Department of Orthopedics, Qingdao the 3rd People’s Hospital, Qingdao, China

**Keywords:** Hyperextension bicondylar tibial plateau fracture, Ligament injury, Popliteal artery, Bone fragment, Hospital for special surgery

## Abstract

**Background:**

Hyperextension bicondylar tibial plateau fracture (HBTPF) is a particular form of tibial plateau fracture which has gained increasing interest recently but were rarely documented. In this study, we reported the characteristics, clinical intervention, and therapeutic outcomes of HBTPF patients.

**Methods:**

From May 2015 to October 2017, clinical data of consecutive patients with bicondylar tibial plateau fractures (BTPF) who underwent surgical treatment in our hospital were retrospectively studied. The patients were allocated to either the HBTPF group (study group) or the non-HBTPF group (control group) based on the radiological features, and inclusion and exclusion criteria. Demographics, characteristics of knee joint injuries, complications, and outcomes were compared between the two groups.

**Results:**

In total, 59 patients were included in this study. Among them, 17 patients with HBTPF were identified and 42 patients were diagnosed as non-HBTPF. No differences in age, sex, cause of injury, side of injury, site of injury, nerve injury, operation time, and treatment time and incision complication between HBTPF and non-HBTPF group. The incidence rate of popliteal artery injury in HBTPF group was 29.4 %, which was significantly higher than that of non-HBTPF group. Small bone chips on the lateral film were found in 94.1 % of the patients in HBTPF group, which was significantly higher than that of non-HBTPF group. The range of motion (ROM) and hospital for special surgery (HSS) score of HBTPF group were significantly lower than those of non-HBTPF group.

**Conclusions:**

HBTPF is a severe injury with a higher incidence rate of popliteal artery injury and worse outcomes than non-HBTPF. Small bone chips at the anterior margin of the proximal tibia on the lateral plain film might be a characteristic of HBTPF.

## Background

Tibial plateau fractures are uncommon injuries. They are more likely to occur among the young population due to high-energy trauma, and due to fragility fractures among the elderly [1]. Their fracture patterns and severity can be characterized by the Schatzker, AO/OTA, or Moore classification systems [2–4]. Bicondylar tibial plateau fractures, or Schatzker V and VI fractures, are considered the most severe tibial plateau fractures. Among them, a subset of injuries caused by hyperextension and high energy mechanisms are usually referred to as hyperextension bicondylar tibial plateau fractures (HBTPF).

HBTPF are complex intra-articular injuries with the presentation of varus, valgus, and pure hyperextension mechanism, leading to injuries in articular congruity, cartilage integrity, and extra-articular structures [2, 5]. In 2016, Firoozabadi et al. first reported the major radiographical changes of HBTPF, including (1) loss of the normal posterior slope of the tibial plateau, (2) tension failure of the posterior cortex, and (3) compression of the anterior cortex due to axial load on a hyperextended knee [6].

The management of HBTPF has been a challenge in clinical setting due to its complexity. Today, the management mainly focuses on two aspects, the restoration of articular congruency and articular alignment, and the soft tissue care and limb survival [7]. Different surgical strategies have been tested to stabilize both the medial and lateral columns for the reconstruction of articular surface and prevention of varus collapse [1, 8]. For example, a meta-analysis showed that highly comminuted bicondylar tibial plateau fractures treated by single lateral locking plate had similar outcomes as dual plate fixation. However, there is very little existing literature regarding the management of soft tissue and associated complications, including compartment syndrome, soft tissue damage, secondary osteoarthrosis (OA), and persistent knee instability [9]. To improve the outcomes of clinical intervention and investigate the complications associated with the HBTPF, we retrospectively studied the HBTPF patients treated in our hospital. The diagnosis and treatment strategy of HBTPF were reported, and treatment outcomes were compared to those of non-HBTPF patients.

## Methods

### Patients

In this retrospective study, clinical data of consecutive patients with bicondylar tibial plateau fracture (BTPF), who were treated in our department from May 2015 to October 2017, were collected. Inclusion criteria were: (1) patients aged 18 years and above; (2) patients examined by X-ray, computed tomography (CT) scan, or magnetic resonance imaging (MRI) with definitive diagnosis of BTPF; (3) has signed the informed consent. Exclusion criteria were: (1) patients with (an)other fracture(s) in addition to BTPF; (2) patient refused to sign informed consent. The study was approved by ethic committee of Tianjin Hospital and was carried out in accordance with the declaration of Helsinki.

### Diagnosis

All patients underwent imaging studies of knee injuries including X-ray, CT scan, and MRI for diagnosis. Diagnostic criteria of HBTPF were (1) sagittal plane malalignment with loss of the normal posterior slope of the tibial plateau, (2) tension failure of the posterior cortex, (3) compression of the anterior cortex [6]. The definitive diagnosis was made jointly by three attending physicians. Patients diagnosed with HBTPF were allocated to the HBTPF group and patients diagnosed with Schatzker type V or VI bicondylar tibial plateau fractures to the non-HBTPF group (control group). Magnetic resonance scanning, although not essential, was carried out to determine associated ligament and meniscus injuries whenever the patient’s condition allowed.

### Treatment

#### Preoperative treatment

The knee line was corrected with calcaneal traction or temporary joint spanning external fixator and the stability of both groups was maintained until definitive fixation was performed. Patients with arterial injuries underwent emergency repair with a great saphenous vein graft, which was conducted by a vascular surgeon, followed by internal fixation or fasciotomy through a temporary knee spanning an external fixator. Anticoagulation therapy with 40 mg low-molecular-weight heparin Q12h was performed daily since admission. Definitive surgery was performed with a thigh tourniquet under general or epidural anesthesia when the condition of the soft tissue had improved.

#### Surgical process

**Fig. 1:**
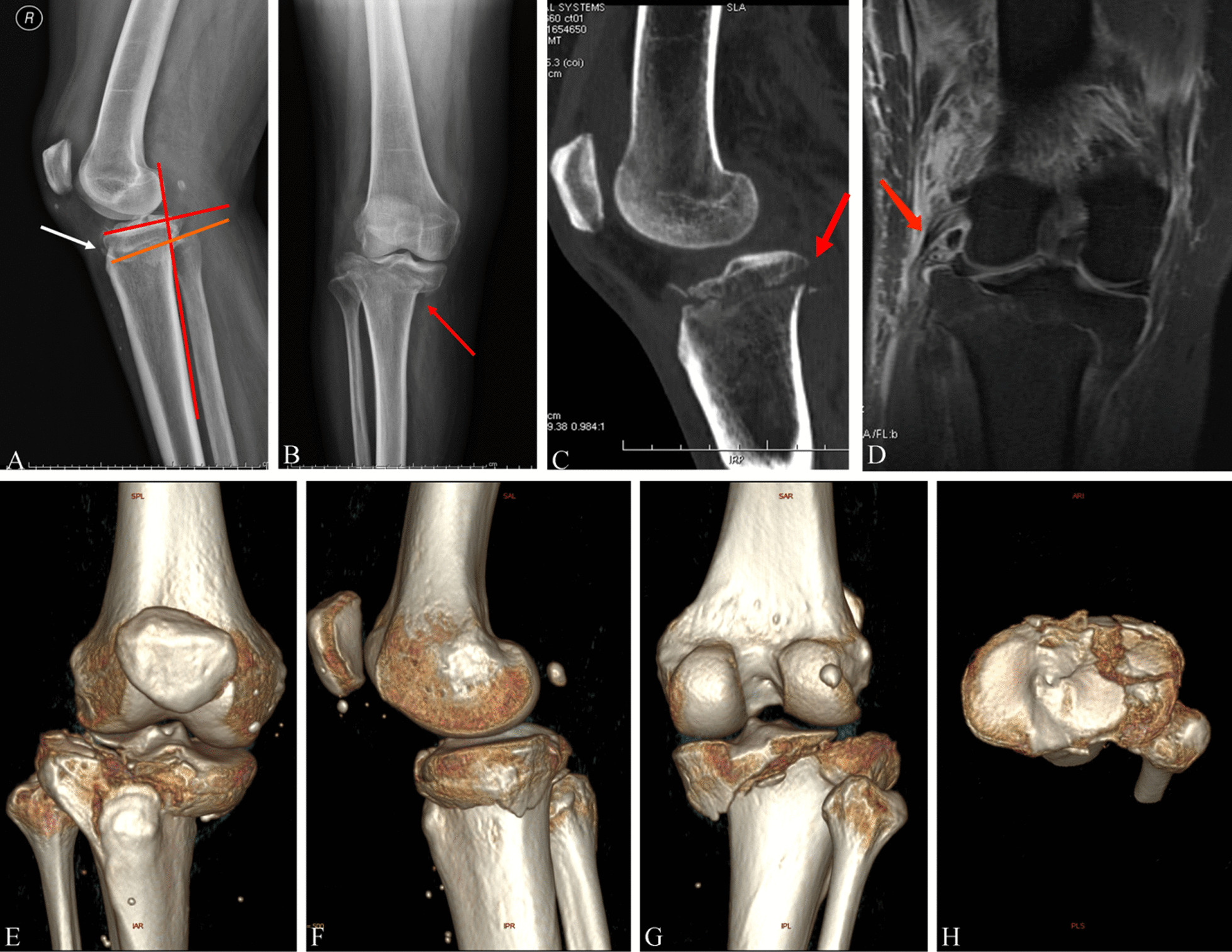
Hyperextension varus bicondylar fracture due to a fall from height. Radiological images of a 59-year-old Chinese male with HBTPF. **a** Red line and orange line showed the reversed slope. White arrow shows the small bone fragment of the fracture. **b** The red arrow showed the anterior compression fracture. **c** Red arrow showed the tension failure. **d**–**h** 3D computed tomography reconstruction preoperatively

**Fig. 2:**
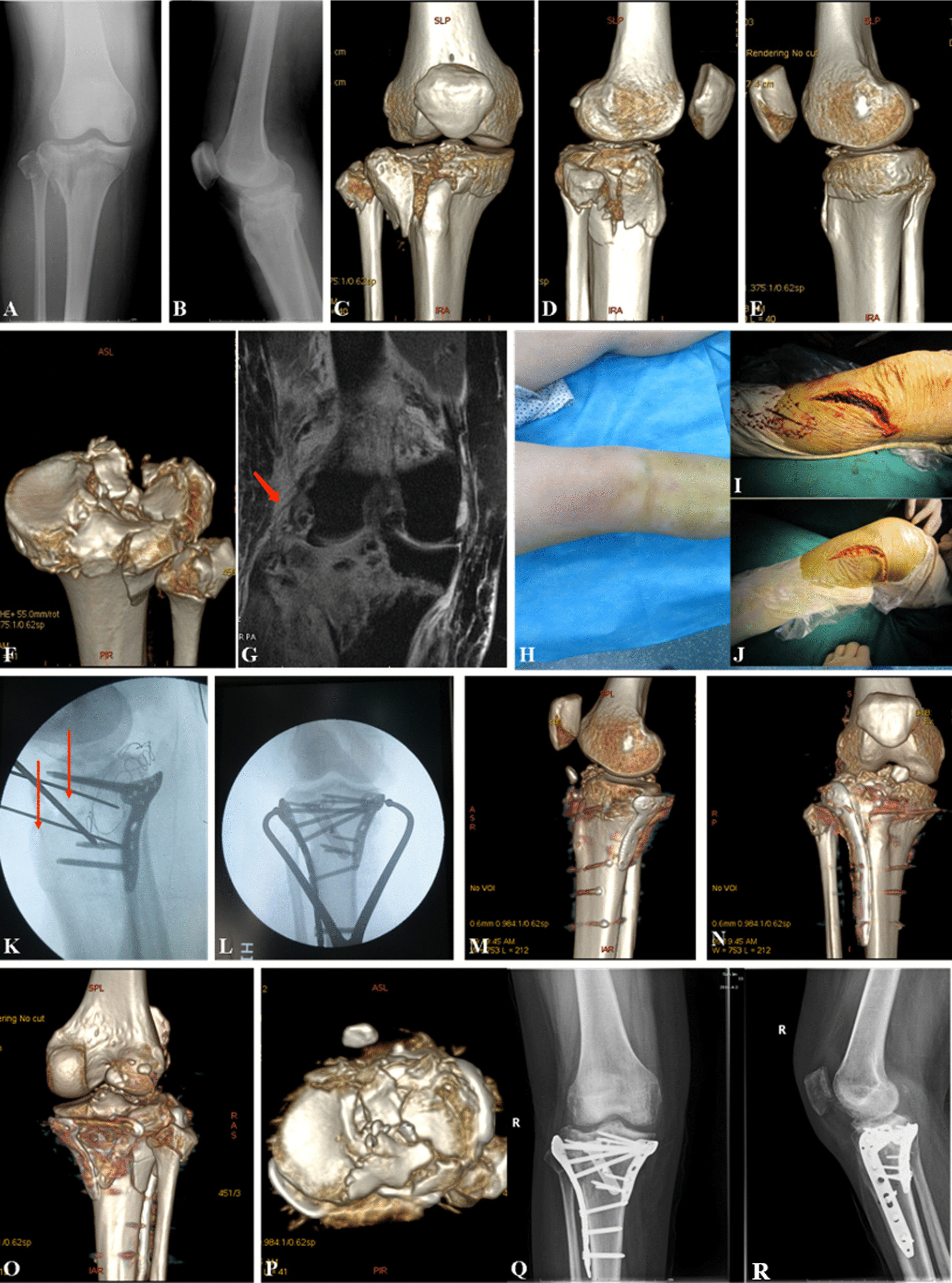
Images of hyperextension bicondylar fracture due to traffic accident. Radiological images of a 50-year-old Chinese male with HBTPF. **a**, **b** X-ray image showed the HBTPF. **c**-**g** 3D computed tomography reconstruction preoperatively. **h** Pre-operative appearance. **i**, **j** showed intraoperative dual incisions. **k **X-ray image, red arrows showed K-wires to reduce the compressed anterior plateau and maintain the reduction. **l** show C-arm. **m-p** 3D computed tomography reconstruction within one week postoperatively. **q**, **r** X-ray images, 7 months postoperatively

**Fig. 3:**
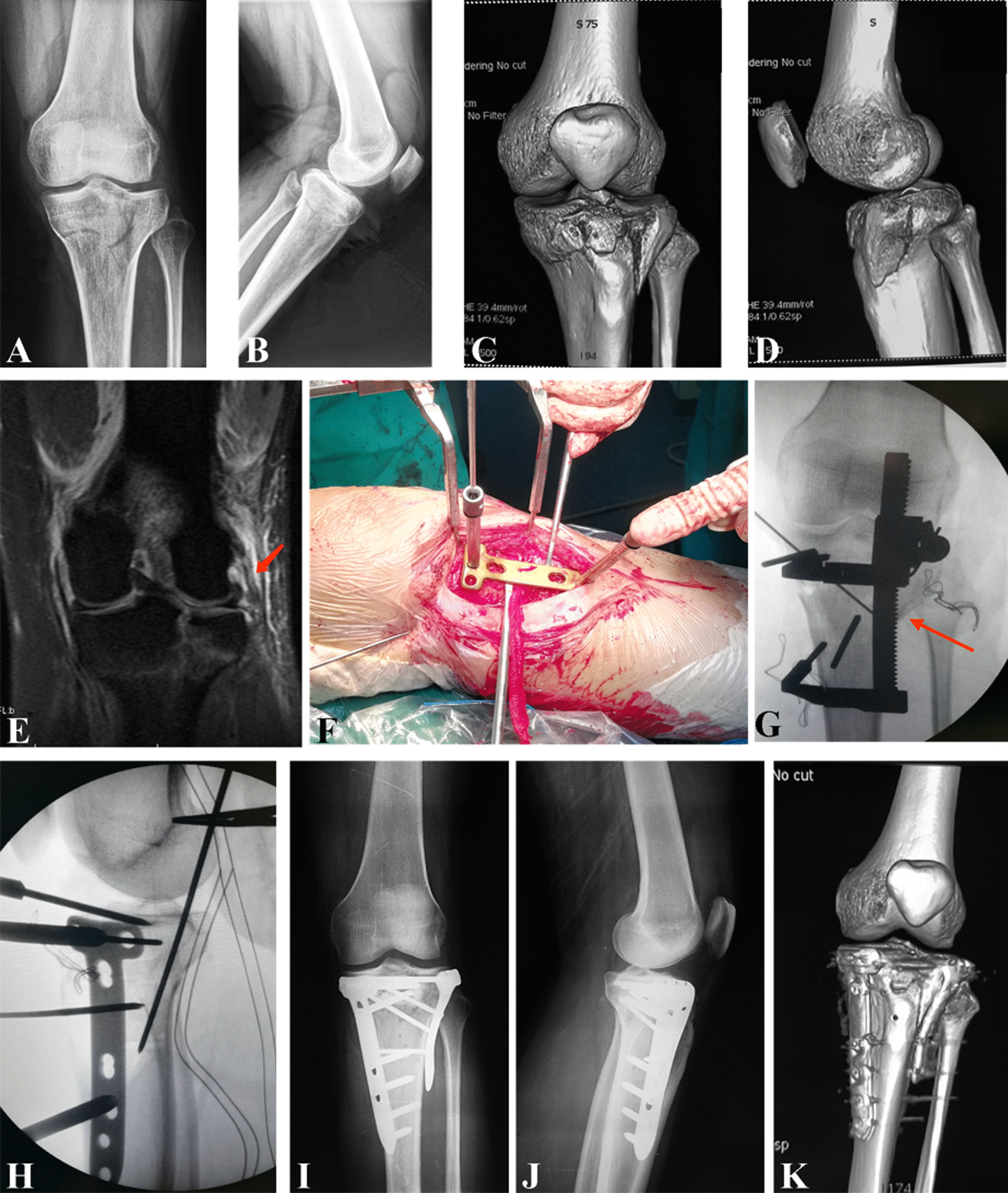
A 36-year old patient with hyperextension bicondylar fracture due to traffic accident. Radiological images of a 36-year-old Chinese female with HBTPF. **a**, **b** X-ray image of injured knee joint preoperatively. **c**, **d** 3D computed tomography reconstruction preoperatively. **e** intraoperative reduction and incisions. **f**, **g** fluoroscopy images of reduction on C-arm. Red arrow in **f** showed the retractor to reduce the compressed anterior plateau and maintain the reduction. **h**, **i** X-ray images within one week postoperatively. **j**, **k** 3D computed tomography reconstruction within 1 week postoperatively

The patient was placed in the supine position with dual incisions made to reduce and fix the fracture with a pre-contoured proximal tibial locking or non-locking plate and screws. Usually, a medial incision is first applied to treat the medial condyle fracture fragment, observe the reduction of its posterior fragment, and protect the pes anserinus tendon during dissection. The medial incisions could be slightly posterior depending on whether the posterior fracture blocks need to be reduced and fixed. For HBTPF patients, after the hematoma and/or fibrous osteotylus were cleaned, the posterior fragment was reduced to a hinge to elevate the compressive anterior fracture. After the blocks and the articular surface were reduced, a locking plate was placed medially, and the placement of the posterior plates (locking or non-locking plates) was dependent on the stability of the posterior fracture fragments, which were exposed through flexion and external rotation of the knee. Based on the reduced medial condyle, the lateral condylar fracture blocks were reduced and fixed with locking plates through a lateral incision. The isolated fracture fragments located at the anterior margin of the proximal tibia were fixed with a 1/3 tubular plate at the operator’s discretion. C-arm fluoroscopy was used to check whether the knee center passed through the lower limb line by stretching the electric knife wire. If the center of the knee was out of line, the fixation needed to be corrected. The bone defect was filled with allogeneic bone or calcium sulfate bone cement. The injured meniscus was repaired or sutured to the capsule during surgery. Meniscectomy is needed if the meniscus was completely damaged and not worth repairing. After fixation of the fracture, a complete collateral ligament rupture was repaired. If knee instability was detected, the posterolateral complex was eventually reconstructed, but a complete cruciate ligament rupture still needed to be repaired arthroscopically. The surgical procedure and preoperative, intraoperative, and postoperative radiographs are shown in Figs. 1, 2 and 3. To control potential biases, the operations and postoperative care were performed by the same senior surgeon and group of nurses, whilst evaluation and data analysis were conducted by other team members.

#### Postoperative care

Rehabilitation exercises were initiated on postoperative day 1. Patients were encouraged to perform quadriceps-strengthening exercises as well as distal limb activity. They were discharged in one or two weeks after surgery and underwent physical therapy during the following 12 weeks.

### Evaluation

After the operation, radiographic examination was required for every patient once a month until the healing of the fracture. After the healing of the fractures, whether the patients complained about any discomfort or not, all patients were followed-up for 24 months. Due to the kind cooperation of the patients, there was no loss to follow-up. Knee function was evaluated during the last clinical visit of patients. The evaluation included range of motion (ROM) and hospital for special surgery (HSS) knee score.

### Statistical analysis

Data were analyzed by SPSS software (version 15.0, IBM, Armonk, NY, USA). Each variable was tested for normality before statistical analysis. Data were presented as mean ± SD (standard deviation) or frequency (%). The difference between the two groups was analyzed by student’s t-test or Chi-square test or Fisher’s exact test where appropriate. All p-values were two tailed and p value less than 0.05 was considered statistically significant.

## Results

### Baseline information

In total, 59 patients were included in this study. 17 patients were diagnosed with HBTPF and 42 patients were diagnosed with BTPF. One patient in the BPTF group had fractures in both knees. Hence, there were 43 fractures in the non-HBTPF group. The average age of the patients were about 50 years, and no difference was found between two groups. More males were found in both groups than females. However, no difference was observed in the distribution of sex in both groups (p = 1.000). The duration from admission to surgery was the same in both groups (p = 0.180). The injuries had four causes, including traffic accident, pedestrian accident, falling, and heavy strike. Among them, traffic accident was the major reason, with about half of the injuries caused by it. It is worth noting that pedestrian accidents were more prevalent in the non-HBTPF group than in the HBTPF group, and patients in the non-HBTPF group were more likely to be injured in the left knees, though no statistical difference was observed. More details were displayed in Table 1.

**Table 1:** Demographic information and clinical characteristics of patients

Variable	HBTPF (n = 17)	BTPF (n = 43)	*p*-value
Age (year)	52.12 ± 9.9	51.21 ± 10.1	0.750
Sex (male/female)	12/5	29/13	1.000
Duration from admission to surgery (day)	11.1 ± 5.5	14.9 ± 9.6	0.180
Cause of injury			0.400
Traffic accident	10 (58.8)	19 (44.2)	
Pedestrian accident	3 (17.6)	18 (41.8)	
Falling	1 (5.9)	3 (7.0)	
Heavy strike	3 (17.6)	3 (7.0)	
Side injured (left/right)	8/9	29/14	0.240
Injury of ligament			
ACL	7 (41.2)	23 (53.5)	0.399
PCL	4 (23.5)	13 (30.2)	0.753
MCL	12 (70.6)	24 (55.8)	0.391
LCL	8 (47.1)	26 (60.5)	1.000
Lateral meniscus	10 (58.5)	24 (55.8)	1.000
Medial meniscus	13 (76.5)	28 (65.1)	0.545
Popliteal artery injury	5 (29.4)	2 (4.7)	0.016
Nerve injury	3 (17.6)	3 (7.0)	0.344
Small bone chips	16 (94.1)	21 (48.8)	0.001

Data were expressed as mean ± SD or frequency (%); *ACL* anterior cruciate ligament, *PCL* posterior cruciate ligament, *MCL* medial collateral ligament, *LCL* lateral collateral ligament

### Clinical characteristics

**Fig. 4:**
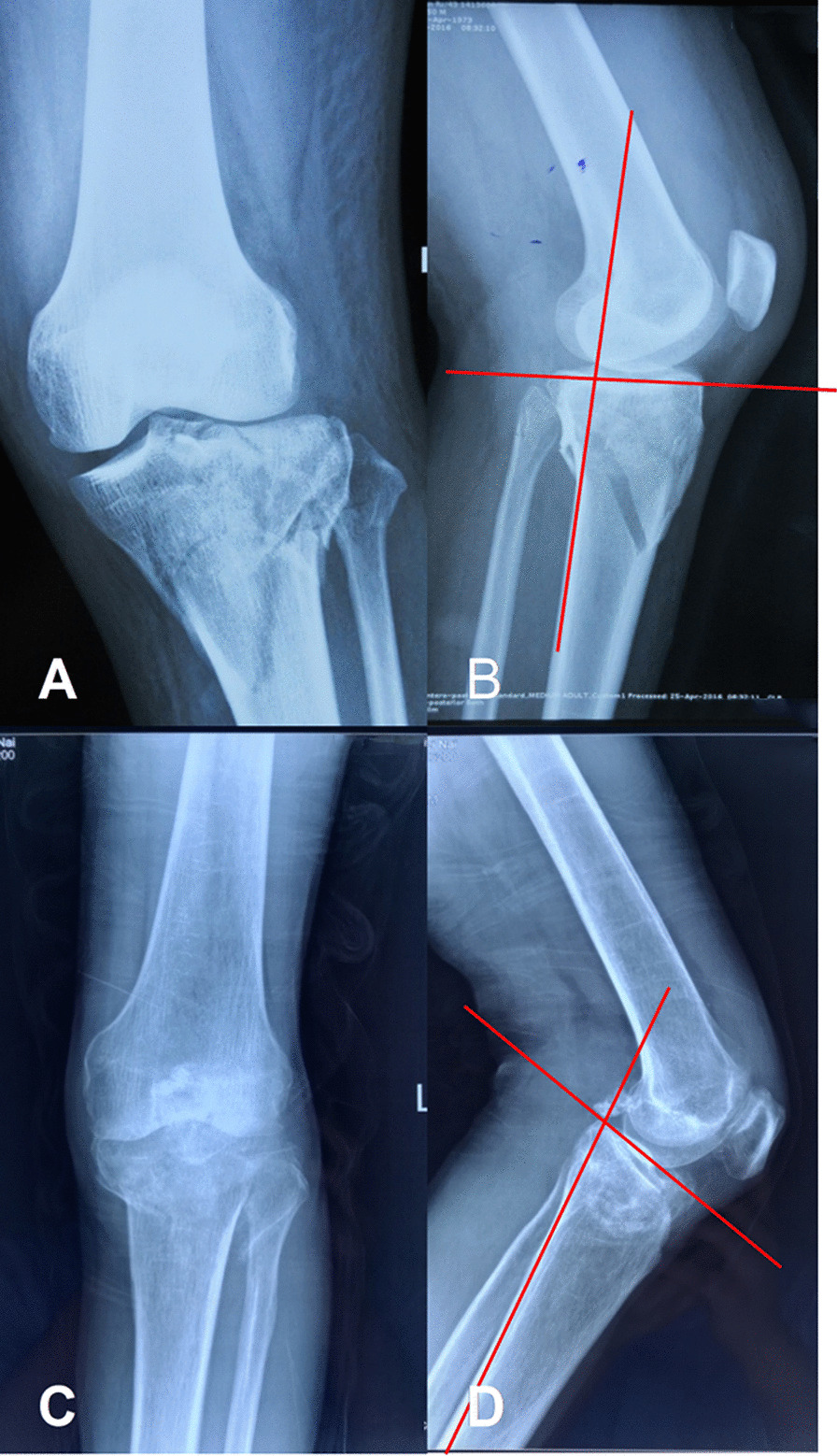
Hyperextensiion and common Schaztker bicondylar tibial plateau fractures. **a** Common Schaztker bicondylar tibial plateau fracture with clear fracture line on the A-P view of X-ray image. **b** Common Schaztker bicondylar tibial plateau fracture with the posterior slope of the tibial plateau on the lateral view of X-ray image. **c** Hyperextension bicondylar tibial plateau fracture (HBTPF), anterior plateau is compressed, and the fracture line is high density on the A-P view of X-ray image. **d** HBPTF, inversed posterior slope of the tibial plateau and distraction rupture of the posterior cortex are observed on the lateral view of X-ray image

The X-ray imaging of HBTPF was characterized by loss or inversion of the posterior slope of the tibial plateau, distraction rupture of the posterior cortex with downward oblique main fracture line, presence of isolated fracture fragment at the anterior margin of the proximal tibia, presence or absence of avulsion fracture of the fibular head on the lateral film, and anterior cortex compression fracture on the coronal image (Fig. 4). These findings are helpful to distinguish HBTPF from Schaztker type V or VI fractures.

Injuries in anterior cruciate ligament (ACL), posterior cruciate ligament (PCL), medial collateral ligament (MCL), and lateral collateral ligament (LCL) were common in both HBTPF and non-HBTPF groups. Though the incidence rates were variable, no statistical difference was found between two groups. In the HBTPF group, the MCL was the ligament that most likely to be injured, with injuries occurring in 70.6 % of the patients. In the non-HBTPF group, the injury rate of ACL, MCL, and LCL were very close, which happened in more than 50 % of the patients. In both groups, the PCL was least likely to be injured (Table 1). Similarly, injuries in the lateral meniscus and the medial meniscus were common in both groups with incidence rates ranging from 55.8 to 76.5 %. No statistical difference was identified. Popliteal artery injuries occurred in both groups. While five out of 17 patients in HBTPF group had popliteal artery injury, only 2 patients in the non-HBTPF group suffered from it. The difference was statistically significant (p = 0.016). Patients who had popliteal artery injuries underwent fasciotomy, except for one patient in the HBTPF group underwent both arterial repair and fracture fixation. Nerve injuries occurred at low levels. There were only 3 patients in each group suffering from peroneal nerve palsy (Table 1). These patients recovered gradually, except for one complained slight numbness at the last clinical visit.

### Clinical outcomes

Forty-two fractures in the non-HBTPF group received definitive fixation. Due to extensive muscle necrosis, one patient underwent amputation. No infection was not observed in HBPTF group, whilst superficial and deep incisional infections were found in 3 and 1 patients respectively in non-HBTPF group. A chronic sinus distal to the incision occurred in one patient and was confirmed to be culture-negative with no effect on fracture healing. Deep vein thrombosis occurred in 10 patients in the HBTPF group and 19 patients in the non-HBTPF group before or after surgery, with no statistical difference between the two groups. All patients recovered in 11 to 18 weeks. In particular, the average bone healing time in both groups were 14.1 and 13.8 weeks in the HBTPF and the non-HBTPF groups, respectively. The difference was not statistically significant. After recovery, the knee joints of HBTPF group showed inferior range of motion (ROM) than that of non-HBTPF group. The HSS score of HBTPF group (89.3 ± 3.5) was lower than that of non-HBTPF group (89.3 ± 3.5), as shown in Table 2. The differences in ROM and HSS between both groups were statistically significant. No loss of sagittal reduction was observed in either group.

**Table 2:** Clinical treatments and outcomes of the patients

Variable	HBTPF (n = 17)	BTPF (n = 43)	*p*-value
Preoperative primary treatment			0.550
None	5(29.4)	13 (30.2)	
Calcaneal traction	9 (52.9)	28 (65.1)	
External fixator	2 (11.8)	2 (4.7)	
Incision complication			
Superficial	0	3 (7.0)	
Deep	0	1 (2.3)	
Sinus tract	0	1 (2.3)	
Deep vein thrombosis	10 (58.8)	19 (44.2)	0.394
Fracture healing time (weeks)	14.1 ± 1.9	13.8 ± 1.8	0.621
Range of motion (°)	120.2 ± 4.6	124.5 ± 7.1	0.041
HSS score	86.9 ± 2.5	89.3 ± 3.5	0.018

Data were expressed as mean ± SD or frequency (%), *HSS* hospital for special surgery

## Discussion

HBTPF has attracted significant interest during the past decade, particularly during the past 5 years [10]. Several reports described anteromedial tibial plateau compression combined with posterolateral corner injury resulted from knee hyperextension varus injury, though the incidence and classification were uncertain [11–13]. One landmark was in 2016 when Firoozadbadi et al. reviewed 25 hyperextension varus bicondylar fractures (11.8 %) among 212 bicondylar fractures over 124 months and recommended the use of the AO/OTA 41C3.3 classification in addition to the Schaztker classification for the classification of these fractures with regard to surgical technique and associated injuries, such as neurovascular complications [6]. Gonzalez et al. reported 15 hyperextension bicondylar fractures (13.0 %) among 115 bicondylar fractures over 93 months [2]. In this study, 17 hyperextension bicondylar fractures among 60 bicondylar fractures were reported over 30 months. The incidence rate of 28.3 % was much higher than what had been reported previously. The main cause of HBTPF was traffic accident, typically where motor vehicles crashed into cyclists.

In this study, we noticed that ligament injuries were associated with the hyperextension fractures, and the incidence rates of anterior cruciate ligament (ALC) and posterior cruciate ligament (PCL) in the non-HBTPF group and the HBTPF group were 41.2 and 23.5 %, respectively. In a cadaveric study, Kennedy found that the ACL was torn first during knee hyperextension, then the posterior capsule and PCL ruptured after 30 degrees of hyperextension, the artery after 50 degrees. In another cadaveric study of knee hyperextension [14], Schenck et al. showed that the fast loading rate of knee hyperextension resulted in PCL avulsions, whereas a slow loading rate resulted in tears in the intermediate region of the PCL, and that the ACL was less sensitive to the loading rate than the PCL [15]. Norwood et al. reported an incident in which the knee was forced into hyperextension, the ACL failure was induced initially by the compression of the intercondylar shelf, and no disruption of the PCL was observed until all of the ACL bundles had failed [16]. We thus presume that the discrepancy between the cadaveric study and this study was due the difference in circumstances. In the cadaveric study, the loading rate, direction, and point of force were all fixed throughout the trials, while in real life, traffic accidents occur randomly; the loading rate, direction, and point of force are neither fixed nor kept the same from crash to crash. It is very difficult to find two traffic accidents identical in these aspects.

To mimic a real-life traffic accident and elucidate the pattern of hyperextension tibial plateau fracture and cruciate ligament injury, Chiba et al. suggested that with ipsilateral feet fixed on the ground, forward inertia and direct impact on the tibia or femur determined the injury pattern under standard procedures [13]. The posterolateral complex (PLC) is an important stabilizing structure of the knee joint, including the lateral collateral ligament, the arcuate ligament, the biceps femoris tendon, the popliteus muscle and tendon, and the fabellofibular ligament. Several studies have reported anterior tibial plateau fractures with PLC injuries, which usually require repair [11–13]. In this study, the PLC injury rate was similar between the two groups, and no complete rupture of other structures was found in the posterolateral complex in addition to complete LCL rupture. However, anteromedial tibial plateau compression fractures were often accompanied by a complete rupture of two or more structures in the PLC. Perhaps the lateral condylar fracture counteracts the entire PLC rupture during the bicondylar fractures, while the hyperextension varus fractures on its own is a diagonal injury. Tomás-Hernández et al. found that small fractures of the anteromedial margin were strongly associated with PCL rupture, whereas the PCL usually remained intact when large fractures occurred. Furthermore, they noted that when the mechanism of hyperextension and forced varus occurred while the PCL was preserved, the impact was generated in the weight-bearing area, leading to anteromedial tibial plateau fracture with large anterior bone fragments. When the PCL was breached, the tibia suffered posterior translation, reducing the impact on the anteromedial weight-bearing area, and producing only a small marginal avulsion [11]. A Segond fracture is usually the result of internal rotation of the knee and varus stress with a motion that creates abnormal tension in the central part of the lateral capsular ligament, often leading to an ACL rupture [17]. However, in our study, only one ACL rupture was found in HBTPF group. It is possible that the energy of the injury was absorbed by the tibial plateau rather than by the cruciate ligament, resulting in fracture rather than ligament rupture.

Arterial injuries are serious limb-threatening injuries during knee injuries, especially in tibial plateau fractures. The annual incidence of popliteal artery trauma was reported to be 2.46 in a population of 10,000 [18]. Tibial plateau fractures have the highest proportion of vascular complications, indicating that immediate therapeutic measures should be taken, while the incidence of popliteal artery injury is even higher in knee hyperextension injuries than that in common injuries [19]. Firoozadbadi et al. reported a 12 % incidence of popliteal artery disruption requiring repair in the hyperextension group compared with 1 % in the control group [6]. In our study, popliteal artery injury occurred in 29.4 % of patients in the HBTPF group and 4.76 % of patients in the non-HBTPF group. Although the incidence of popliteal artery injury in this study was lower than that reported by Firoozadbadi, the difference between HBPTF and non-HBPTF was statistically significant. Popliteal artery injury during knee injury may result from the compression of the fractured fragments, direct blunt injury, and distraction injury. The distraction injury may develop into extensive vascular endothelial damage, which leads to vascular inactivity. It is worth noting that HBTPF is associated with a higher risk of popliteal artery injury. In this study, no patient in the HBTPF group was amputated, while one in the control group was amputated due to failure in popliteal artery repair. The restoration of the repaired artery depends on many factors, such as the condition of the vascular bed, the degree of vascular injury, and the duration of distal limb ischemia collateral circulation.

A noteworthy feature of HBTPF is that small bone chips were frequently observed at the anterior margin of the tibia on the lateral plain film. Segond fracture also occurs at the tibial insertion site of the anterolateral ligament. Therefore, there is a very low chance of generating any small bone chips on the lateral plain film. Thus, the small bone fragments observed in our study differ from the Segond fractures. Chiba et al. suggested that the small chips were due to the compression of the femoral condyle during knee hyperextension and varus [13]. However, in this study, only 7 out of 16 patients with small bone chips in the HBTPF group showed evidence of femoral condyle injury. We speculate that the small bone chips were from two sources. One is the compression of the femoral condyle, in which case, the small bone chips coexist with the femoral condyle injury. Another is that the small bone chips detached from the anterior proximal tibia when the elastic articular cartilage impinges on the hard-tibial tubercle. This may explain why only seven patients showed signs of damage to the femoral condyle.

For the treatment of HBPF, conservative treatment may have a poor prognosis due to compression fractures, reversed joint, and extensive soft tissue damage. Therefore, surgical treatment of HBPF is recommended. However, reported surgical results are poor. Firoozabadi et al. reported 25 cases of surgical treatment of varus HBTPF with a high incidence of associated injuries, compartment syndrome, and neurovascular injuries with complications including one nonunion of total knee arthroplasty, two wound infections, one wound dehiscence, one hematoma requiring debridement, two radiographic medial arthropathies, one deep vein thrombosis, and one removal of symptomatic internal fixation [6]. However, they did not report the functional outcome scores. Gonzalez et al. reported that patients with hyperextension bicondylar fracture had worse knee function compared with the controls [2], similar to the results obtained by Wu et al., who suggested that more extensive damage to knee ligaments and soft tissue may account for the poor outcome of the HBTPF because more scars during the healing process could limit the flexion and extension of the knee joint [20]. In our study, the values of the range of motion and HSS scores indicated that the knee function of the HBTPF group was inferior than that of the non-HBTPF group.

This study has several limitations. First, the small sample size and single-center study cannot objectively represent the general treatment outcomes of the hyperextension fractures. Furthermore, patients requiring amputation in emergencies were excluded. Therefore, the true incidence rate of the artery and nerve injury was not obtained. Due to a short follow-up time, secondary osteoarthrosis (OA) and long-term treatment results remain unknown.

## Conclusions

Compared to non-HBTPF, HBTPF was less common and usually associated with more arterial injuries. Small bone chips were frequently observed at the anterior margin of the proximal tibia, which could be one of the features of HBPTF. Even after surgical intervention, the prognosis of HBTPF was worse than that of non-HBPTF.

## Data Availability

The datasets used and/or analyzed during the current study are available from the corresponding author on reasonable request.

## Abbreviations

ACL: Anterior cruciate ligament
BTPF: Bicondylar tibial plateau fracture
HBTPF: Hyperextension bicondylar tibial plateau fracture
HSS: Hospital for special surgery
MCL: Medial collateral ligament
LCL: Lateral collateral ligament
PCL: Posterior cruciate ligament
PLC: Posterolateral complex
ROM: Range of motion

## References

[1] Metcalfe D, Hickson CJ, McKee L, Griffin XL (2015). External versus internal fixation for bicondylar tibial plateau fractures: systematic review and meta-analysis. J Orthop Traumatol.

[2] Gonzalez LJ, Lott A, Konda S, Egol KA (2017). The hyperextension tibial plateau fracture pattern: a predictor of poor outcome. J Orthop Trauma.

[3] Marsh JL, Slongo TF, Agel J, Broderick JS, Creevey W, DeCoster TA (2007). Fracture and dislocation classification compendium – 2007: Orthopaedic Trauma Association classification, database and outcomes committee. J Orthop Trauma.

[4] Moore TM, Patzakis MJ, Harvey JP (1987). Tibial plateau fractures: definition, demographics, treatment rationale, and long-term results of closed traction management or operative reduction. J Orthop Trauma.

[5] Jansen H, Frey SP, Doht S, Fehske K, Meffert RH (2013). Medium-term results after complex intra-articular fractures of the tibial plateau. J Orthop Sci.

[6] Firoozabadi R, Schneidkraut J, Beingessner D, Dunbar R, Barei D (2016). Hyperextension varus bicondylar tibial plateau fracture pattern: diagnosis and treatment strategies. J Orthop Trauma.

[7] Chouhan DK, Chand Saini U, Kumar Rajnish R, Prakash M (2020). Complex bicondylar tibial plateau fractures with reversed tibial slope - Our experience with a fracture-specific correction strategy. Trauma Case Rep.

[8] Chang H, Zhu Y, Zheng Z, Chen W, Zhao S, Zhang Y (2016). Meta-analysis shows that highly comminuted bicondylar tibial plateau fractures treated by single lateral locking plate give similar outcomes as dual plate fixation. Int Orthop.

[9] De Coster TA, Nepola JV, El-Khoury GY. Cast brace treatment of proximal tibia fractures. A ten-year follow-up study. Clin Orthop Relat Res. 1988(231):196–204. 3370874

[10] Zhao R, Lin Z, Long H, Zeng M, Cheng L, Zhu Y (2019). Diagnosis and treatment of hyperextension bicondylar tibial plateau fractures. J Orthop Surg Res.

[11] Tomás-Hernández J, Monyart JM, Serra JT, Vinaixa MR, Farfan EG, García VM (2016). Large fracture of the anteromedial tibial plateau with isolated posterolateral knee corner injury: case series of an often missed unusual injury pattern. Injury.

[12] Conesa X, Minguell J, Cortina J, Castellet E, Carrera L, Nardi J (2013). Fracture of the anteromedial tibial plateau associated with posterolateral complex injury: case study and literature review. J Knee Surg.

[13] Chiba T, Sugita T, Onuma M, Kawamata T, Umehara J (2001). Injuries to the posterolateral aspect of the knee accompanied by compression fracture of the anterior part of the medial tibial plateau. Arthroscopy.

[14] Gustafson RC, Petreman MC (1963). Complete dislocation of the knee joint. Can Med Assoc J.

[15] Schenck RC, Kovach IS, Agarwal A, Brummett R, Ward RA, Lanctot D (1999). Cruciate injury patterns in knee hyperextension: a cadaveric model. Arthroscopy.

[16] Norwood LA, Cross MJ (1977). The intercondylar shelf and the anterior cruciate ligament. Am J Sports Med.

[17] Gottsegen CJ, Eyer BA, White EA, Learch TJ, Forrester D (2008). Avulsion fractures of the knee: imaging findings and clinical significance. Radiographics.

[18] Ramdass MJ, Muddeen A, Harnarayan P, Spence R, Milne D (2018). Risk factors associated with amputation in civilian popliteal artery trauma. Injury.

[19] Ottolenghi CE. Vascular complications in injuries about the knee joint. Clin Orthop Relat Res. 1982;165:148–156. 7075053

[20] Wu K, Huang J, Lin J, Wang Q (2017). Diagnosis and treatment of anterior tibial plateau fracture-dislocation: a case series and literature review. J Knee Surg.

